# Preparation of Graphene Oxide-Embedded Hydrogel as a Novel Sensor Platform for Antioxidant Activity Evaluation of *Scutellaria baicalensis*

**DOI:** 10.3389/fchem.2021.675346

**Published:** 2021-04-16

**Authors:** Shuai Yan, Yinzi Yue, Li Zeng, Lianlin Su, Min Hao, Wei Zhang, Xiaopeng Wang

**Affiliations:** ^1^State Key Laboratory of Quality Research in Chinese Medicine, Macau University of Science and Technology, Macau, China; ^2^Suzhou TCM Hospital Affiliated to Nanjing University of Chinese Medicine, Suzhou, China; ^3^First Clinical Medical College, Nanjing University of Chinese Medicine, Nanjing, China; ^4^School of Pharmacy, Nanjing University of Chinese Medicine, Nanjing, China; ^5^School of Pharmacy, Zhejiang Chinese Medicine University, Hangzhou, China

**Keywords:** antioxidant activity, *Scutellaria baicalensis*, electrochemical analysis, hydrogel, graphene oxide

## Abstract

Antioxidation is very important in medicine and food. The current evaluation technologies often have many shortcomings. In this work, an improved electrochemical sensing platform for the evaluation of antioxidant activity has been proposed. A hydrogel was prepared based on graphene oxide, zinc ions, and chitosan. Zinc ions play the role of crosslinking agents in hydrogels. The structure of chitosan can be destroyed by injecting hydrogen peroxide into the hydrogel, and the free zinc ions can diffuse to the surface of the electrode to participate in the electrochemical reaction. This electrochemical sensor can evaluate the antioxidant activity by comparing the current difference of zinc reduction before and after adding the antioxidant. With the help of graphene oxide, this hydrogel can greatly enhance the sensing effect. We conducted tests on 10 real samples. This proposed electrochemical platform has been successfully applied for evaluating the antioxidant activity of *Scutellaria baicalensis*, and the results were compared to those obtained from the 2,2-diphenyl-1-picrylhydrazyl-based traditional analysis technique.

## Introduction

Antioxidants are substances that effectively inhibit the oxidation of free radicals when present in low concentrations. They can capture excess free radicals, neutralize them, and reduce the damage caused by oxidative stress. Antioxidants can effectively clean up excess free radicals in the body and prevent various diseases (Apak et al., 2018; Moteshakeri et al., 2018; Aghdam et al., 2019; Karimi-Maleh et al., 2021a). Antioxidants can be divided into exogenous antioxidants and endogenous antioxidants. Among them, exogenous antioxidants are the antioxidants that are taken into the human body through food, such as flavonoids, vitamins, hormones, phenolic acids, and esters (Brainina et al., 2019; Karimi-Maleh et al., 2019; Masek et al., 2019; Kurtulbaş et al., 2020).

Many studies have proved that Chinese herbal medicine has antioxidant active ingredients that can clean up and reduce the damage of the free radicals in the organism (Han et al., 2017; Jin et al., 2018; Malekmohammad et al., 2019). At the same time, Chinese herbal medicine can also effectively enhance immunity. According to the main antioxidant components present, the antioxidants in Chinese herbal medicine can be divided into phenylhexoside, ginsenoside, flavonoids, alkaloids, anthraquinone, and polysaccharides (Karimi-Maleh et al., 2021b). However, evaluating the antioxidant activity of Chinese herbal medicine is still a difficult problem.

So far, the main methods used to evaluate antioxidant activity include the hydrogen atom transfer method, the single-electron transfer method, the chromatographic method, and the electrochemical method (Shabani et al., 2020; Ye et al., 2020; Carp et al., 2021). These methods all have some disadvantages. For example, the hydrogen atom transfer method is when the antioxidants transfer hydrogen atoms to free radicals, making them inactive (Xu et al., 2020; Zhang et al., 2020; Zhou et al., 2020). The advantage of this method is that it can be used to determine water-soluble and oil-soluble substances, but the disadvantage is that the light probe is sensitive and the method is time-consuming (Boudier et al., 2012). The reaction rate of the single-electron transfer method is usually very low, and it takes a long time to complete the detection. Chromatography allows quantification of the antioxidant capacity of OH, H_2_O_2_, and peroxynitrite. However, this method is not easily adaptable for high-throughput analysis requiring quality control, because it requires multiple injections to measure the ethylene production (Shui and Leong, 2004; Cimpoiu, 2006).

On the other hand, electrochemical methods have attracted a lot of attention because of their rapidity and efficiency (Kilmartin, 2001; Blasco et al., 2007; Teixeira et al., 2013; Apak et al., 2016). The non-radical electrochemical methods include cyclic voltammetry, enzyme voltammetry, and potentiometric analysis (Ghanei-Motlagh et al., 2019; Ghanei-Motlagh and Baghayeri, 2020; Naderi Asrami et al., 2020; Karimi-Maleh et al., 2021c; Nodehi et al., 2021). Electrode poisoning is the reduction of efficiency on the electrode surface due to the deposition of reactants (Karimi-Maleh et al., 2020). Recently, an electrochemical method based on hydrogel has been used for evaluating the antioxidant activity (Fu et al., 2018a,b). This method uses the signal from the metal ions in the hydrogel linkage, which could provide sensitive determination of the antioxidant activity in the whole electrolyte system. In this work, we further developed an advanced hydrogel using chitosan, graphene oxide (GO), and zinc ions. The presence of GO in the hydrogel system accelerates the electron transfer rate of the metal ions during the sensing, which improves the performance of the platform. *Scutellaria baicalensis* Georgi has been used as a real example for evaluating the practical application of the proposed method.

## Materials and Methods

All materials used in this work were analytical grade. Zinc acetate, acetic acid, ascorbic acid, ascorbic acid, 2,2-diphenyl-1-picrylhydrazyl (DPPH), and gallic acid were purchased from Aladdin Reagent Inc (Chuhuazhi Rd, Shanghai, China). Chitosan (50,000–190,000 Da) was purchased from Sigma-Aldrich (St. Louis, MO, United States). Graphite powder was purchased from Linke ChemTech Co. Ltd. (College Street, Kolkata, India). The morphology of the hydrogel was observed using a field emission scanning electron microscope (FESEM, Apreo, FEI). The x-ray diffraction (XRD) patterns of the sample were collected using an X-ray diffractometer (Broker Philips PW1730). Thermal gravimetric analysis (TGA) was carried out using a TGA instrument (BÄHR-Thermoanalyse GmbH, Altendorfstraße 12 D-32609, Hüllhorst). The Fourier-transform infrared spectroscopy (FTIR) spectra of the sample were characterized using an FTIR spectrophotometer (Bruker vector FTIR).

Preparation of the hydrogel: GO was prepared using a typical Hummers' method (Chen et al., 2013). The prepared GO was dispersed into water to form a 0.5 mg/ml dispersion for further use. For the synthesis of the hydrogel, a certain amount of GO dispersion was added into 10 ml of 1 wt% chitosan solution (in 1% acetic acid) under stirring. Then, 0.1 ml of zinc acetate solution was added as well. After half an hour of stirring, NaOH (0.1 M) was added drop-by-drop until the initiation of the gelation process. The formed hydrogel was denoted as C/GO/Zn. The hydrogel without GO was prepared using a similar process without the addition of GO dispersion. This product was denoted as C/Zn.

Preparation of *Scutellaria baicalensis* extract: A 5 g sample of *Scutellaria baicalensis* powder was weighed and 70% ethanol was added in the mass ratio of 1:30 (powder:solvent). The ultrasonic-assisted extraction method was used for extraction three times, 20 min each time. After filtration, the supernatant was taken as the extract.

Antioxidant activity test: the antioxidant activity test was carried out using two methods. The first one is the DPPH measurement. Typically, 5 mg DPPH was dissolved in 125 ml of C_2_H_5_OH. Then, 0.8 ml of DPPH solution was mixed with 0.2 ml of the *Scutellaria baicalensis* extract or different concentrations of tocopheryl, and the reaction was kept away from light for 15 min at room temperature. Then, the UV-Vis spectrophotometer was used to determine the spectrophotometry value of the solution, which was determined to be at 514 nm. The second method is the hydrogel-based electrochemical method. Typically, after the addition of the *Scutellaria baicalensis* extract or different concentrations of tocopheryl, a certain amount of H_2_O_2_ solution was injected. Sonication was conducted for 30 s to accelerate the diffusion. Then, a glassy carbon electrode (GCE), Ag/AgCl (3M), and a Pt wire were inserted into the hydrogel. Either cyclic voltammetry (CV) or differential pulse voltammetry (DPV) was used for measuring the redox of the Zn ions for evaluating the antioxidant activity of the samples.

## Results and Discussion

**Figure 1:**
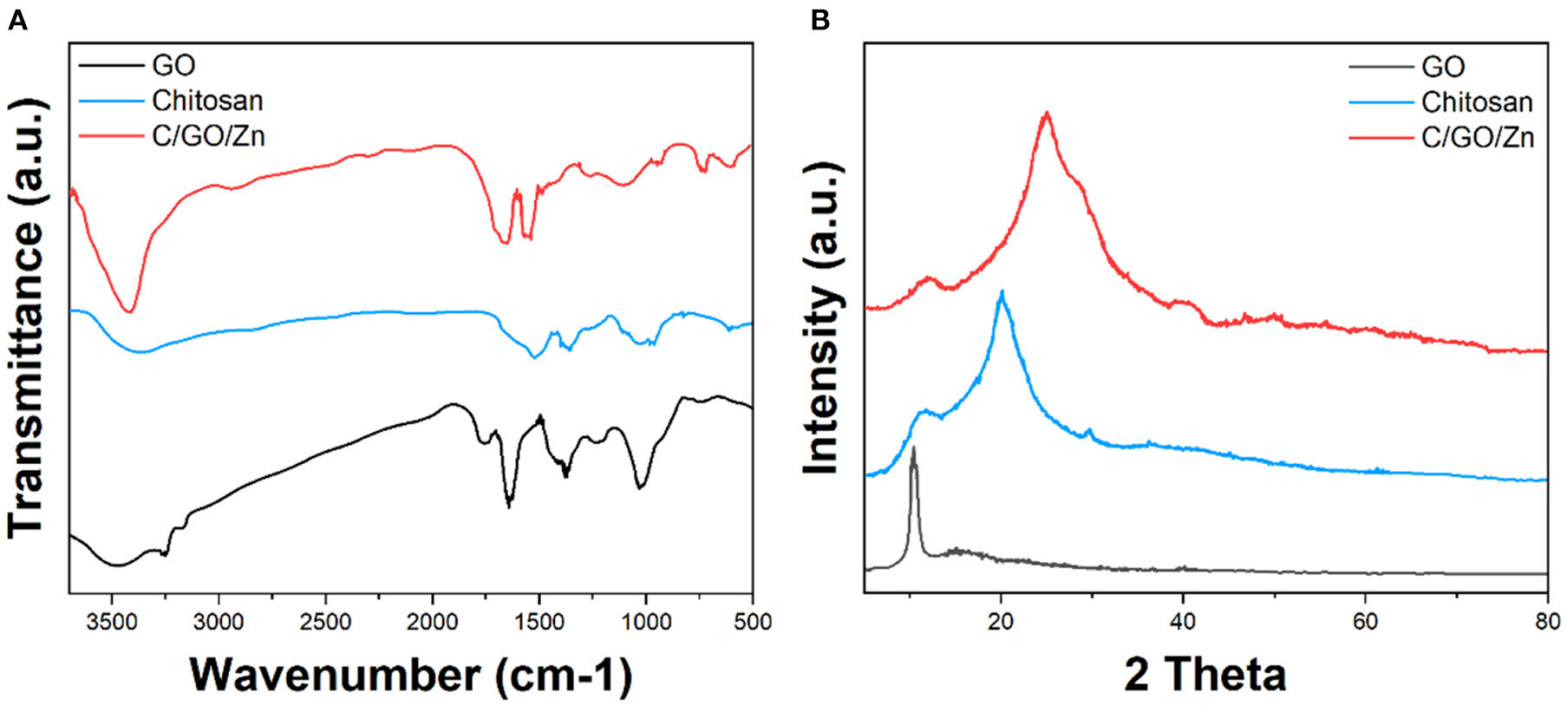
**(A)** FTIR spectra and **(B)** XRD patterns of GO, chitosan, and C/GO/Zn.

Figure 1A shows the FTIR spectra of GO, chitosan, and C/GO/Zn. As shown in the figure, the spectrum of GO shows the peaks located at 1,042, 1,626, 1,725, and 3,397 cm^−1^, which can be assigned to the stretching vibrations of the C-O, C=C, C=O, and O-H bonds (Strankowski et al., 2016), respectively. The presence of the peaks at 1,042 and 3,397 cm^−1^ indicate the successful formation of GO. The spectrum of chitosan shows a series of peaks between 1,750 and 600 cm^−1^, indicating the stretching of the C-H, C=O, C=C bonds (Manoratne et al., 2017), while the peaks between 3,000 and 3,500 cm^−1^ can be ascribed to the stretching of the OH group (Lawrie et al., 2007). In addition, the spectrum of C/GO/Zn shows the combination of both the materials, indicating the successful formation of the composite in the hydrogel.

Figure 1B shows the XRD pattern of GO, chitosan, and C/GO/Zn. The pattern of GO shows a typical peak at around 11° due to the plane (200) of GO (Stobinski et al., 2014). The chitosan shows a series of peaks between 10° and 30° due to the polymeric networks (Tang et al., 2003). The C/GO/Zn shows a very similar pattern compared with that of the chitosan. However, we can still observe the appearance of the peak corresponding to the plane (200) of GO.

**Figure 2:**
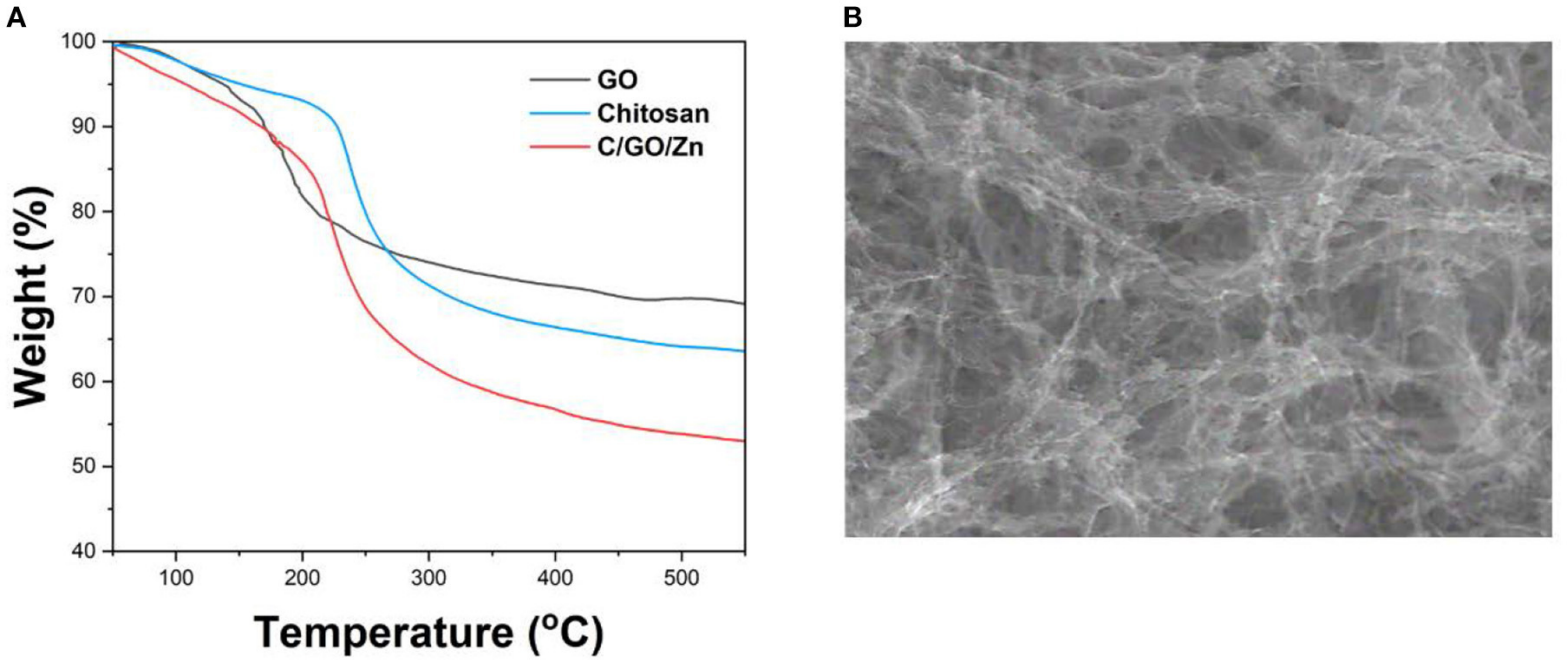
**(A)** TGA curves of GO, chitosan, and C/GO/Zn. **(B)** SEM image of C/GO/Zn.

Figure 2A shows the TGA profiles of GO, chitosan, and C/GO/Zn. It can be seen that the GO shows a poor thermal property, which causes weight loss at <100°C. This weight loss can be ascribed to the evaporation of water. Then, the GO shows a fast decline loss between 150 and 200°C, indicating the reduction of oxygen-containing groups of the sheets plane (Li et al., 2012). The curve of the chitosan shows that the mass loss begins at 230°C and continues until 400°C. It can be due to the depolymerization of chitosan and the degradation of glycosidic units. The further decline of the weight above 400°C can be ascribed to the breakdown of the structure of chitosan (Corazzari et al., 2015). The curve of C/GO/Zn shows that the main weight loss starts at above 205°C. The decline of the thermal stability of the hydrogel can be ascribed to the formation of a network between the two materials. The poor stability of the GO lowers the thermal stability of the hydrogel.

Figure 2B shows the Scanning electron microscopic (SEM) image of the C/GO/Zn hydrogel. It can be seen that the hydrogel shows a very porous structure. This structure can be ascribed to the successful formation of cross-links between zinc ions and chitosan. In addition, the presence of GO in the hydrogel network can be clearly identified.

**Figure 3:**
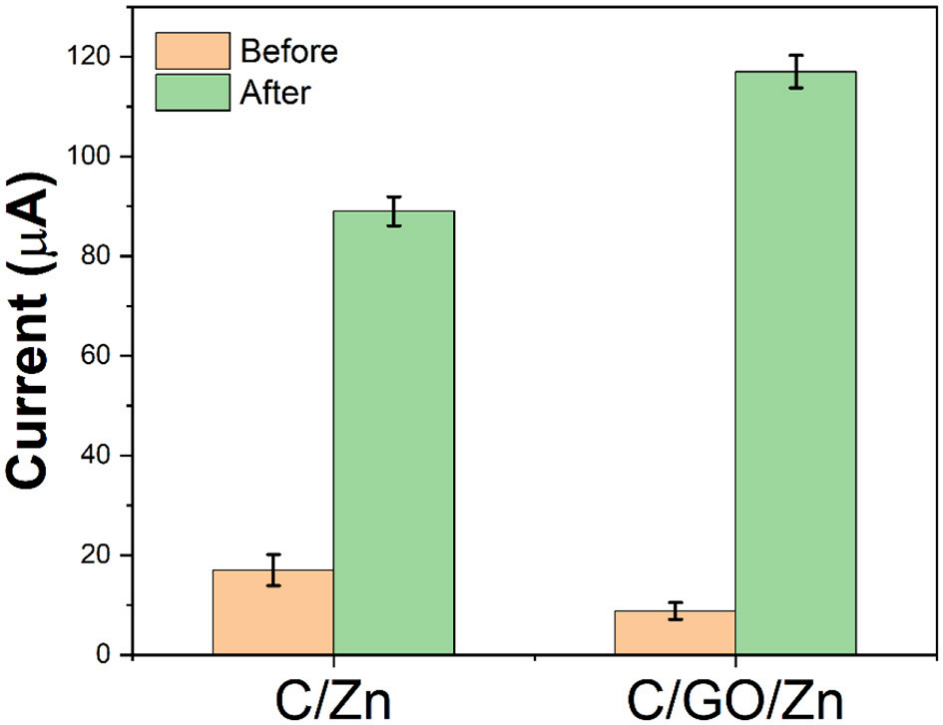
Current value of C/Zn and C/GO/Zn before and after the injection of 0.1 ml H_2_O_2_ (10%) after 10 min.

Since chitosan needs to be dissolved in an acetic acid solution, an acetic acid buffer solution was selected for the preparation of the sensor platform. In the C/GO/Zn hydrogel, zinc ions are used as crosslinking agents to connect chitosan and GO. Therefore, if electrochemical scanning is carried out in the hydrogel, the Zn ion will not be able to move freely from the hydrogel to the electrode surface, hence, the electrochemical response will be low. After the injection of hydrogen peroxide, the free zinc ions in the hydrogels become more diverse due to the slow destruction of the structure of chitosan by the hydrogen peroxide. These free zinc ions can rapidly diffuse to the electrode surface to participate in the redox reaction. We use −0.6 V as the reduction potential of the zinc ions and reflect the content of free zinc ions in hydrogels according to the current value. At the same time, by comparing the current values before and after the injection of hydrogen peroxide, we can evaluate the damage effect of free radicals on chitosan. It can be seen from Figure 3 that without GO, a certain amount of hydrogen peroxide produces a current difference of 72 μA. However, with the participation of GO, the same amount of hydrogen peroxide produces a current difference of 109 μA. There are two reasons for this increase in the current difference. First, GO helps the electron transfer of the zinc ions. The second reason is that GO affects the stability of hydrogels, so that more zinc ions will be released in the C/GO/Zn hydrogel under the injection of the same amount of hydrogen peroxide.

**Figure 4:**
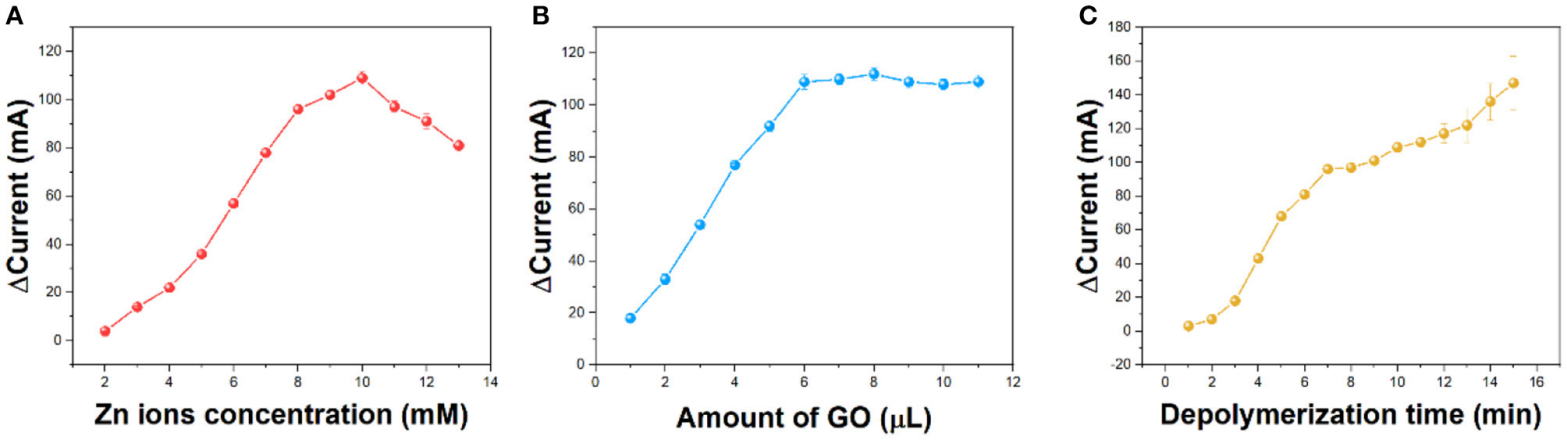
Effect of **(A)** concentration of Zn ions, **(B)** amount of GO dispersion, and **(C)** depolymerization time on the current difference recoded from CV.

Zinc ion addition is a very important factor. If the amount of zinc added is not enough, the hydrogel will not be formed. If there are too many zinc ions, the free zinc ions in the hydrogel will affect the accuracy of the detection. Figure 4A shows the effect of different zinc ion concentrations on hydrogels. It can be seen from the figure that zinc ions increase the corresponding current from 2 to 10 mM. Above 10 mM, the difference in the value of current begins to decrease. Therefore, we chose 10 mM zinc ions for the preparation of hydrogels.

The addition of GO is also a very important factor. GO in fewer amounts will not be able to enhance the detected signal. By contrast, too much GO will hinder the formation of the hydrogels. As can be seen from Figure 4B, the current intensity increases from 1 to 6 μl and reaches the saturation point. Increasing the amount of GO will only have a small impact. If the amount of GO is more than 11 μl, the C/GO/Zn hydrogel will not be formed. Therefore, 6 μl of GO has been selected for preparation.

The waiting time for the hydrogen peroxide to depolymerize is also important. Sufficient time for depolymerization time can make enough zinc ions participate in the electrochemical reduction. As shown in Table 1, the reduction current gradually increases from 1 to 10 min and remains stable. Although increasing the depolymerization world can further increase the current, these tests have large errors. Therefore, we chose 10 min as the depolymerization time.

**Table 1:** The antioxidant capacity of 10 samples of *Scutellaria baicalensis* detected using the DPPH method and the proposed electrochemical method.

**Sample No**.	**DPPH (mgTrolox/mg)**	**Electrochemical method (μA)**
1	46.21 ± 1.07	40.1
2	37.44 ± 0.78	29.8
3	36.51 ± 2.21	35.2
4	42.01 ± 1.04	33.5
5	44.28 ± 1.22	31.6
6	36.89 ± 1.50	24.7
7	50.36 ± 1.42	22.2
8	31.04 ± 0.94	42.1
9	39.98 ± 0.71	37.5
10	41.20 ± 1.03	36.9

**Figure 5:**
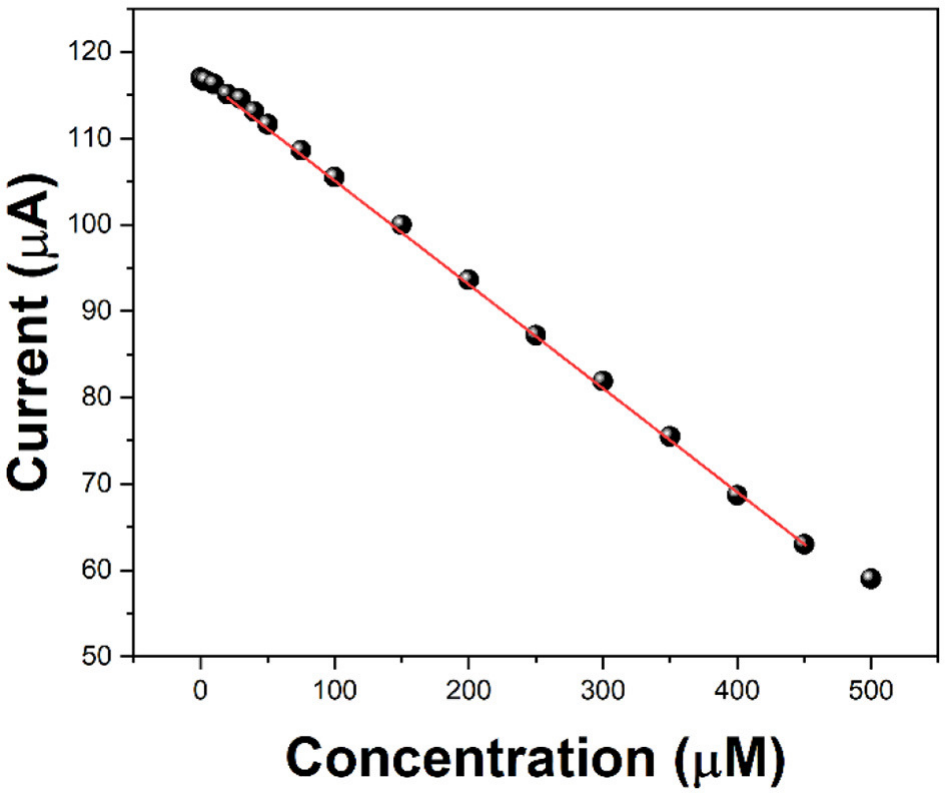
Current value of C/GO/Zn after the injection of 0.1 ml of H_2_O_2_ (10%) and different concentrations of ascorbic acid.

Ascorbic acid is used as a detection molecule to measure the antioxidant activity evaluation performance of the electrochemical platform. After 5 min of hydrogen peroxide injection, we added different concentrations of ascorbic acid, and then tested it at 10 min. Because ascorbic acid prevents the free radicals from attacking the chitosan molecules further, the reduction current of zinc ions will be reduced. Compared with the current value without ascorbic acid and with ascorbic acid, it can be used to evaluate the antioxidant activity of ascorbic acid. Figure 5 shows the current values against the addition of 0.5 to 500 μM of ascorbic acid. It can be seen that the increase in the concentration of ascorbic acid lowers the current response. It can be seen that in the range of 2–450 μM, the decrease of the current value presents a linear relationship, so it can be used to evaluate the antioxidant activity of ascorbic acid.

We tested the antioxidant activity of *Scutellaria baicalensis* purchased from 10 different samples. In addition to the electrochemical detection method proposed in this paper, the DPPH method was also used for comparison. In DPPH detection, Trolox was used as the reference material. The antioxidant capacities of these 10 samples were calculated by measuring the absorbance of different concentrations of Trolox and DPPH at the same time. The results are shown in Table 1. It can be seen from the table that the antioxidant capacities of the 10 samples of *Scutellaria baicalensis* are not the same. This may be because of the fact that they come from different places of origin or use different processing technologies. Although the two detection techniques cannot be compared horizontally, it can be seen from the comparison of the differences between the samples that the results of the proposed electrochemical detection method are basically consistent with those of the DPPH detection method. In both detection techniques, sample 7 shows the strongest antioxidant activity, while sample 8 shows the weakest.

## Conclusion

In this work, we proposed an electrochemical platform based on a hydrogel synthesized using graphene, chitosan, and zinc ions. This electrochemical platform can be used for evaluating the antioxidant capacity by monitoring the current change with the reduction of zinc ions. The addition of GO significantly enhances the current response, which could be used for high-sensitivity sensing. The proposed electrochemical platform was successfully used for evaluating the antioxidant capacity of *Scutellaria baicalensis*.

## Data Availability Statement

The original contributions presented in the study are included in the article/supplementary material, further inquiries can be directed to the corresponding authors.

## Author Contributions

SY, WZ, and XW conceived of the study. XW and WZ supervised the development program. SY, MH, and YY collected materials characterization. YY and LZ received and curated samples and analytical records. LS, MH, and SY wrote the manuscript. All authors read and approved the manuscript.

## Conflict of Interest

The authors declare that the research was conducted in the absence of any commercial or financial relationships that could be construed as a potential conflict of interest.
